# Multicenter Evaluation of Decentralized Homologous Recombination Deficiency Testing for Enhanced Molecular Diagnosis of Ovarian Cancer ^†^

**DOI:** 10.3390/ijms272110028

**Published:** 2026-11-09

**Authors:** Gardenia Vargas-Parra, Marta Sesé, Eva Hernández-Illán, Laia Serrano, Laura Camacho, Rosa Somoza, Vanessa López, Cristina Teixido, Pedro Jares, Javier Hernández-Losa, Beatriz Bellosillo

**Affiliations:** 1 Pathology Department, Hospital del Mar Research Institute, Barcelona 08003, Spain; lserranomunne@hmar.cat (L.S.); lcamacho@hmar.cat (L.C.); 2 Centro de Investigación Biomédica en Red de Cáncer (CIBERONC), ISCIII, Madrid 08028, Spain; 3 Pathology Department, Hospital Universitari Vall d’Hebron, Barcelona 08035, Spain; marta.sese@vallhebron.cat (M.S.); javier.hernandez@vallhebron.cat (J.H.-L.); 4 Translational Molecular Pathology, Vall d’Hebron Institut de Recerca, Barcelona 08035, Spain; rosa.somoza@vhir.org; 5 Molecular Biology CORE, Hospital Clínic de Barcelona, Barcelona 08036, Spain; evhernandez@clinic.cat (E.H.-I.); vlopez3@clinic.cat (V.L.); teixido@clinic.cat (C.T.); pjares@clinic.cat (P.J.); 6 Pathology Department, Hospital Clínic de Barcelona, Barcelona 08036, Spain; 7 August Pi i Sunyer Biomedical Research Institute (IDIBAPS), University of Barcelona, Barcelona 08036, Spain

**Keywords:** homologous recombination deficiency, high-grade serous ovarian carcinoma, *BRCA1*, *BRCA2*, genomic instability, PARP inhibitors, low-pass whole-genome sequencing, molecular diagnostics

## Abstract

Assessment of homologous recombination deficiency (HRD) and tumor *BRCA1/2* mutation status in high-grade serous ovarian carcinoma (HGSOC) is clinically relevant for treatment decisions and for identifying tumors that may benefit from poly(adenosine diphosphate–ribose) polymerase inhibitors. The Myriad MyChoice^®^ CDx HRD (M-HRD) assay is an established clinically validated HRD testing platform that is performed as a send-out test. We conducted a multicenter pilot study to assess the analytical feasibility and interlaboratory agreement of decentralized testing using the SOPHiA DDM™ Dx HRD Solution (SG-HRD). Three hospitals each contributed eight clinical HGSOC samples previously tested with M-HRD or with SG-HRD in a send-out platform. All centers independently prepared SG-HRD libraries and sequenced the complete set of 24 samples. Agreement with M-HRD was evaluated separately from comparisons with previous SG-HRD send-out results. Among specimens with interpretable M-HRD, overall percent agreement with local SG-HRD results was 95.0% (19/20), 90.0% (18/20), and 88.9% (16/18) at the three hospitals. *BRCA1/2* variant calls were concordant with previous testing. Overall, the comparison with previous analyses by Myriad genetics or with previous SOPHiA GENETICS send-out results showed a high level of agreement, with discordances mainly observed in samples with values close to the classification threshold. The small, selected cohort, limited clinical replication, and absence of treatment-outcome assessment restrict precision estimates, generalizability, and conclusions regarding clinical validity. These findings support the analytical feasibility of decentralized SG-HRD testing, but larger studies are needed to characterize performance across a broader range of specimens and testing conditions.

## 1. Introduction

This article is a revised and expanded version of a poster entitled “Multicenter study to validate and implement SOPHiA DDM HRD solution for enhanced molecular diagnosis of ovarian cancer”, which was presented at the 2024 European Society of Gynaecological Oncology Congress in Barcelona, Spain [1].

High-grade serous ovarian carcinoma (HGSOC) remains the leading cause of mortality among gynecological malignancies in developed countries, largely due to late-stage diagnosis and high rates of disease recurrence [2,3].

Several established and inherited risk factors contribute to the development of HGSOC including age, hormonal and reproductive factors, a strong family history of breast or ovarian cancer (OC) and other hereditary predisposition syndromes [4]. Among these, germline pathogenic variants in the *BRCA1* (BReast CAncer gene 1) and *BRCA2* (BReast CAncer gene 2) genes are the most well-established genetic risk factors conferring a substantially increased lifetime risk of OC. The cumulative risk of developing OC by age 70 is estimated at 40–44% for *BRCA1* mutation carriers and 17–18% for *BRCA2* mutation carriers [5].

Beyond their role in hereditary cancer predisposition, BRCA1 and BRCA2 are key components of the homologous recombination repair (HRR) machinery, a high-fidelity DNA repair mechanism that preserves genomic stability by enabling the accurate repair of DNA double-strand breaks and replication-associated lesions. BRCA1 participates in DNA-end processing and coordinates pathway choice toward homologous recombination. PALB2 functions as a molecular scaffold linking BRCA1 to BRCA2, while BRCA2 facilitates the loading and stabilization of RAD51 nucleoprotein filaments on resected single-stranded DNA. RAD51-mediated strand invasion subsequently enables homology-directed DNA synthesis using the sister chromatid as a template. These proteins also contribute to the protection and stabilization of stalled replication forks, thereby limiting the accumulation of replication-associated genomic damage [6].

Loss of function (LoF) variants in *BRCA1/2, BRCA1* promoter methylation, and other alterations affecting the HRR pathway, may impair these processes and lead to homologous recombination deficiency (HRD) [7]. In HRD cells, DNA lesions are increasingly repaired through more error-prone mechanisms, resulting in chromosomal rearrangements, copy-number alterations and progressive loss of genomic integrity. These changes leave characteristic genomic scars, including loss of heterozygosity (LOH), telomeric allelic imbalance (TAI) and large-scale state transitions (LSTs). Genomic scars are historical footprints of defective DNA repair rather than direct measurements of the current functional state of the HRR pathway. Their accumulation provides an indirect record of previous HRR deficiency and can therefore be used as a biomarker of the HRD phenotype [8].

The genomic instability resulting from HRD contributes to tumor evolution, but it is also associated with increased sensitivity to poly(adenosine diphosphate–ribose) polymerase inhibitors (PARPi) and other DNA-damaging agents across several cancer types, including breast, prostate, pancreas and OC [9,10,11,12]. PARP1 and PARP2 participate in the detection and signaling of DNA single-strand breaks and other forms of DNA damage. Pharmacologic PARP inhibition suppresses PARP catalytic activity and can stabilize PARP-DNA complexes (“PARP trapping” on chromatin), thereby increasing replication-associated DNA lesions. In cells with functional HRR, these lesions can be resolved through homologous recombination; in HRD cells, these lesions cannot be efficiently repaired, resulting in replication fork collapse, chromosome instability and, ultimately, cell death through a synthetic lethal interaction. Approximately 50% of HGSOC tumors harbor HRD [3]. The use of reliable methods to establish tumor HRD status is of critical importance for the management of advanced HGSOC, serving as a predictive biomarker for therapeutic benefit from PARPi maintenance therapy and platinum-based chemotherapy [13].

HRD can be assessed through the combined analysis of the three main genomic scars described above [8]. SNP array-based technologies, such as OncoScan^TM^ CNV (ThermoFisher Scientific, USA), Infinium CytoSNP-850K (Illumina, USA) or Affymetrix SNP 6.0 (Affymetrix, USA), evaluate genomic instability by profiling copy number alterations (CNAs) and chromosomal aberrations across the genome. The data generated are analyzed using specific algorithms to derive any of the three HRD genomic scars [14,15]. In contrast, functional approaches such as nuclear RAD51 foci quantification assesses active DNA repair capacity in S/G2 phase cells, rather than recording historical genomic footprints [16].

The U.S. Food and Drug Administration (FDA) has granted approval for HRD testing through commercial send-out platforms. These assays assess *BRCA1*/2 mutational status and genes involved in the HRR pathway, together with at least one of the three HRD genomic scars. Tempus xT CDx (Tempus AI, Inc., USA), the most recently approved test for tumor-only indication [17], and FoundationOne^®^ CDx and Foundation One^®^ Liquid CDx for cell-free DNA analysis (Foundation Medicine, USA) focus on genomic LOH and microsatellite instability (MSI). In contrast, Myriad MyChoice^®^ CDx HRD (hereafter referred to as M-HRD) (Myriad Genetics; USA) evaluates all three genomic scars [10,18,19]. Although liquid biopsy-based approaches are increasingly being investigated for HRD assessment, this remains an evolving field, and their clinical utility and analytical concordance with tissue-based assays continue to be evaluated.

Additional decentralized commercial panels have been developed for the study of HRD-associated genomic scars. AmoyDx^®^ HRD Focus Panel (Amoy Diagnostics, Singapure) (hereafter referred to as AmoyDx HRD) estimates a “genomic scar score” using a machine learning method which predicts HRD events from CNAs present in the analyzed genomic region [20]. Oncomine™ Comprehensive Assay Plus (commonly known as OCA+) (ThermoFisher Scientific, USA) and TruSight™ Oncology 500 HRD (TSO 500 HRD) (Illumina, USA) both analyze more than 500 genes and the three HRD genomic scars [21,22].

There are also whole-genome sequencing (WGS) and whole-exome sequencing (WES) classifiers such as HRDetect, CHORD and other bioinformatic packages [23,24,25] and academic solutions like the Leuven HRD Test, combining genome-wide SNP profiling and exons of 9 HRR genes [26] and the NOGGO GIS Assay combining the analysis of 20,000 SNPs and 57 genes [27], which have been created to reduce turnaround times and testing costs.

Cost-effective low-pass WGS (~0.3× to 1× coverage) techniques focus on genome-wide large-scale genomic alterations and CNAs analysis. SeqOne HRD Solution (Agilent Technologies, USA) uses low-pass WGS at ~0.3× and a 16 gene panel [28] and SOPHiA DDM™ Dx HRD Solution (SOPHiA GENETICS SA, Switzerland) (hereafter referred to as SG-HRD) combines low-pass WGS (~1×) for genome-wide assessment of chromosomal alterations with targeted deep sequencing of genes involved in the HRR pathway. Genome-wide copy number and allelic patterns are computationally integrated into a genomic integrity (GI) index using a machine-learning-based analytical framework. The GI index provides a continuous measure of genomic instability, which is subsequently interpreted together with *BRCA1/2* and other HRR-gene alterations to determine the molecular HRD classification. SG-HRD stands out as one of the HRD assessments most concordant with the FDA-approved M-HRD [29,30].

Differences in tumor cellularity, sample quality, assay design, bioinformatic pipelines, thresholds, and the detection of germline versus somatic alterations can also affect HRD classification [31,32]. These analytical and biological differences may lead to discordant results between platforms and underscore the importance of validating decentralized assays against clinically established reference methods before their implementation in routine diagnostic workflows.

While centralized send-out testing offers some benefits like specialized equipment, standardized procedures and potentially lower costs due to scalability, it also poses several challenges for routine patient diagnosis, like transportation expenses, sample logistical issues, time delays, administrative overhead or limited accessibility for clinicians to comment on secondary results. Moreover, the use of external testing may result in healthcare resources being transferred outside the local health system.

Previous studies have provided analytical and clinical evidence supporting the GIInger-based approach used by the SOPHiA HRD solution. Pozzorini et al. demonstrated analytical concordance with an established comparator and, in a retrospective analysis of 195 patients from the PAOLA-1 trial, identified a GI-positive subgroup with significantly longer progression-free survival with olaparib plus bevacizumab than with placebo plus bevacizumab [33]. More recently, Rojo et al. reported the use of the SOPHiA HRD solution in 4777 tumor samples within a five-center diagnostic network in Spain, providing large-scale evidence of its implementation in clinical practice [34]. These findings provide a rationale for further evaluating agreement when testing is performed independently across laboratories.

In the present multicenter pilot study, we aimed to compare in-house SG-HRD classification of HGSOC samples previously tested with the FDA-approved M-HRD assay, at three hospitals, to support the implementation of SG-HRD as a decentralized testing method. Evaluating the diagnostic concordance between decentralized approaches and a clinically validated comparator is important before the in-house implementation in routine pathology workflows.

## 2. Results

### 2.1. Sample Quality and Sequencing Metrics

The 24 clinical samples selected for the study were processed for HRD and *BRCA1/2* assessment. All DNA samples met the requested quality criteria for library preparation with the SG-HRD solution. The percentage of mapped reads was >91% for all samples and all samples reached 100× coverage across the regions of interest.

Sequencing metrics for both the targeted HRR-gene panel and low-pass WGS component met predefined quality requirements in most samples across the three participating centers. However, eight samples processed at hospital 3 yielded a lower number of reads. Five of them were flagged as low-quality. Despite these lower sequencing metrics, 21 of 24 samples yielded evaluable GI indices, and *BRCA1/2* results were successfully generated for all samples. Detailed sequencing and quality metrics are summarized in the Table S1.

### 2.2. HRD Scoring and Variant Calling

Overall, comparison of the in-house SG-HRD results with previous clinical HRD results, including both M-HRD analyses and SG-HRD send-out testing, showed a high level of agreement. To assess variant calling performance, we compared the results obtained at the three centers with those obtained in prior send-out testing. No discrepancies were identified in *BRCA1/2* variant calls relative to prior testing among the 24 clinical samples (Table 1). In addition, all commercially defined *BRCA1/2* variants included in the SeraSeq control were correctly detected in both intra- and inter-run analyses across all centers.

Using the evaluable-sample population, HRD scoring concordance between the current in-house SG-HRD results and the corresponding previous send-out result was 95.5% (21/22), 86.4% (19/22), and 85.0% (17/20) for hospitals 1, 2, and 3, respectively.

Discordant HRD classifications were observed in samples 13, 18 and 20. Sample 13 represented a borderline case, with a previous SG-HRD GI index of 0 and values ranging from −0.4 to 0.0 across the participating centers. Sample 20 similarly showed values close to the positivity threshold, ranging from −0.7 to 1.5. One additional discordant HRD result was observed between the previous analysis and the results obtained in the present study. In this case (sample 18), the previous analysis had been performed on a biopsy, whereas the current study used a surgical specimen from the same patient and organ. This difference in sample source may have contributed to the discordant classification.

We then assessed HRD classification concordance specifically against M-HRD. Two samples of the selected cohort for this work (samples 11 and 13) had been previously assessed using SG-HRD and were therefore not considered when specifically evaluating concordance with M-HRD as the clinical comparator. When the analysis was restricted to samples with a previous M-HRD result, HRD overall percent agreement (OPA) between in-house SG-HRD and M-HRD was 95.0% (95% CI:75.1–99.9%) (19/20) at Hospital 1, 90.0% (95% CI:68.3–98.8%) (18/20) at Hospital 2, and 88.9% (95% CI:65.3–98.6%) (16/18) at Hospital 3. The corresponding Cohen’s ĸ values were 0.898, 0.798 and 0.775, respectively. For Hospital 3, this analysis was restricted to the 18 samples with an evaluable GI index.

With M-HRD as the clinical comparator, positive percent agreement (PPA) was 100.0% (11/11; exact 95% CI, 71.5–100.0%), 90.9% (10/11; 58.7–99.8%), and 90.0% (9/10; 55.5–99.7%) at Hospitals 1, 2, and 3, respectively. Negative percent agreement (NPA) was 88.9% (8/9; 51.8–99.7%), 88.9% (8/9; 51.8–99.7%), and 87.5% (7/8; 47.3–99.7%), respectively. The paired counts (SG-HRD+/M-HRD+, SG-HRD+/M-HRD−, SG-HRD−/M-HRD+, SG-HRD−/M-HRD−) were (11, 1, 0, 8), (10, 1, 1, 8), and (9, 1, 1, 7).

### 2.3. Repeatability and Reproducibility

The study design enabled assessment of intra-run repeatability, inter-run reproducibility, and inter-center concordance. Sample 1 was analyzed in duplicate within Run1 (A-1 and A-2) and independently in Run 2 (A-3). Across these replicate measurements, *BRCA1*/2 variant detection remained concordant and the sample was consistently classified as HRD-positive. GI index values ranged from 3.9 to 5.7 across the three participating centers and sequencing runs, indicating stable genomic-instability measurements. The replicate and control-material experiments documented consistent results under the conditions tested. However, the clinical replicate experiment involved only one HRD-positive specimen and did not characterize precision across the range of GI values, tumor content, DNA quality, variant allele frequencies, or positive and negative clinical specimens.

Intra-run assessments showed high repeatability of variant detection and variant allele fraction (VAF) measurements, with observed variability remaining within the theoretical expectations based on high DNA input and ~200× mean coverage. All detected variants showed VAF above the predefined analytical thresholds of 4% for single-nucleotide variants and insertions/deletions, supporting consistent variant detection above the predefined analytical threshold.

Inter-run evaluations similarly demonstrated stable variant calls and VAF values across independent sequencing runs and closely matching expected values within the calculated 1σ and 3σ deviation ranges.

In addition, the independent analysis of the complete clinical sample set by the three participating hospitals enabled assessment of inter-center reproducibility. Pairwise agreement in GI-based classification was 91.7% between Hospitals 1 and 2 (22/24; exact 95% CI, 73.0–99.0%), 90.5% between Hospitals 1 and 3 (19/21; 69.6–98.8%), and 100.0% between Hospitals 2 and 3 (21/21; 83.9–100.0%). Comparisons involving Hospital 3 excluded samples 6, 9, and 10 because no evaluable GI index was available. The principal differences among hospitals occurred in samples close to the predefined HRD classification threshold.

## 3. Discussion

In this multicenter study, we aimed to assess the feasibility of conducting HRD testing in-house using samples that had been previously analyzed with a validated test for HRD assessment (M-HRD). The assay showed complete concordance for *BRCA1/2* variant detection and high consistency in HRD classification.

HRD is an established predictive biomarker of sensitivity to platinum-based treatment and PARPi in ovarian cancer. From a molecular perspective, HRD assays provide complementary information at different biological levels. Pathogenic alterations in *BRCA1/2* and other HRR-related genes may identify a potential cause of defective DNA repair, whereas genomic instability scores capture the cumulative chromosomal consequences of HRR dysfunction. These consequences include TAI, LOH and LST that accumulate during tumor evolution. Therefore, genomic scar-based approaches can identify an HRD-associated phenotype in tumors without detectable *BRCA1/2* pathogenic variants, as observed in several samples included in the present study. This is clinically relevant because HRD extends beyond *BRCA1/2*-mutated ovarian cancer.

The best way to ensure that a test is accurate is to validate it clinically, based on the response to targeted treatment. Although our study was not designed to directly assess treatment response, comparison with a clinically validated reference method provides relevant evidence supporting the potential clinical implementation of the decentralized assay. Clinical validation of SG-HRD as a biomarker for PARPi response was conducted on a subset of 384 FFPE OC samples from the PAOLA-1 clinical trial. Retrospective testing with SG-HRD classified 98.4% of samples for HRD status. Among patients with HRD-positive tumors, median progression-free survival (PFS) was 35.7 months longer in the olaparib plus bevacizumab arm than in the placebo plus bevacizumab arm (HR 0.36, 95% CI 0.25–0.51, *p* < 0.001) whereas no significant PFS difference was observed in the HRD-negative group (HR 1.02, 95% CI 0.71–1.48, *p* = 0.90) [30].

Local NGS panels provide an accessible alternative for estimating genomic instability without requiring sample transfer to central reference laboratories. Cross-validation studies demonstrate strong agreement with the reference assay. The AmoyDx HRD assay displayed a linear correlation coefficient of r = 0.79 for numerical scar scores and an OPA of 88.6% (95% CI: 75.4–96.2%) when benchmarked against M-HRD in 50 epithelial OC tissue samples [35]. In line with these findings, a German interlaboratory comparison reported categorical concordance exceeding 94% for both NOGGO GIS and the TSO 500 HRD platform [36]. Similarly, OCA+ demonstrated 90.9% OPA in 55 cytological samples from HGSOC when compared with matched histological samples previously tested using M-HRD [37]. Additional evidence supporting the performance of decentralized HRD assays was provided by the MITO16A/MaNGO-OV2 study, in which DNA from 100 treatment-naïve OC patients was assessed using M-HRD as the reference and three alternative NGS-based methodologies: TSO 500 HRD, OCA+ and SG-HRD. All three assays showed high concordance with M-HRD. *BRCA* mutation agreement ranged from 94.4% to 97.7%, genomic instability from 88.2% to 95.3%, and HRD status from 90.4% to 97.6%. HRD-positive patients had similar response rates, PFS, and overall survival outcomes to those observed with the M-HRD [38].

Alternative genomic architectures deliver comparable diagnostic accuracy. Traditional SNP microarrays, such as OncoScan™ CNV, demonstrated strong continuous correlation (r = 0.87) and an OPA ranging from 77.5% to 83.0% relative to M-HRD [35]. Concurrently, low-pass WGS has emerged as a cost-effective strategy [38]. Multiple evaluations of low-pass WGS pipelines support these results, consistently maintaining OPA rates between 90 and 95% across the 24 and 20 HGSOC samples previously evaluated with M-HRD by Jinga et al. and Scaglione et al., respectively [39,40].

Other studies have also compared the agreement between SG-HRD and other HRD testing strategies. A significant correlation between M-HRD and SG-HRD results was found in the study of Guarishi-Sousa et al., where 85 OC samples were tested with two different NGS methods (in-house SG-HRD and Amoy HRD testing) [29]. Also, the study of Pepe et al. assessed HRD status in 20 OC samples using three commercially available in-house NGS-based assays (SG-HRD, Amoy HRD focus and Oncomine HRR pathway predesigned panel) conducted across three different laboratories, each of them using a different methodology. The overall agreement between the three was 76.5%, and SG-HRD exhibited the highest agreement (90%) with the M-HRD test in categorizing samples as HRD-positive or HRD-negative [41].

Fellowes et al. also evaluated SG-HRD against M-HRD in a larger cohort of 145 OC samples obtained from four laboratories and reported 100% concordance for *BRCA1/2* mutation status and 91.2% OPA after optimizing the GI positivity threshold from 0 to −1.5, with discordances around the clinical cutoff [42]. In the present work, including 58 evaluable comparisons, the SG-HRD assay showed high concordance (91.4%) (53/58) with M-HRD and interlaboratory agreement across the three independent hospital laboratories. Complete concordance was observed for *BRCA1/2* variant detection, while GI index results were highly consistent among centers, with the limited discrepancies mainly affecting samples close to the positivity threshold. Interestingly, if the −1.5 GI threshold identified by Fellowes et al. had been applied to our dataset, concordance would have increased to 94.8%. A distinctive feature of our study was the analysis of the same complete set of 24 samples independently at each of the three participating hospitals, rather than different samples subsets at each center. This paired multicenter design allowed direct assessment of interlaboratory concordance while keeping the biological composition of the test set constant, providing a controlled evaluation of the reproducibility of decentralized testing.

Notably, although eight samples processed at Hospital 3 yielded lower read counts and five were flagged as low quality, reduced sequencing yield selectively impacted the low-pass WGS component, resulting in unavailable GI index values in 3/8 samples, whereas deep targeted sequencing retained sufficient analytical performance for *BRCA1*/2 variant detection. These findings suggest that the two sequencing components of the assay may differ in their susceptibility to reduced sequencing yield, while preservation of *BRCA1/2* variant detection supports the analytical feasibility of the assay under suboptimal sequencing conditions.

The limited discordances observed in our study were mainly concentrated in samples with GI index values close to the predefined positivity threshold. This finding is biologically and analytically plausible because genomic instability represents a continuous phenotype, whereas clinical assays translate it into a binary HRD-positive or HRD-negative classification. Consequently, small differences in tumor-cell content, DNA quality, library balance, tissue sampling or subclonal composition may shift a borderline result across the decision threshold. This was illustrated by samples with GI index values ranging from −0.7 to 1.5 across centers, despite otherwise similar molecular results. In addition, the discordant classification observed in sample 18 may have been influenced by sample provenance; the previous analysis was performed on a biopsy whereas the present study used a surgical specimen from the same patient. Spatial intratumoral heterogeneity and differences in subclonal representation may therefore contribute to discordant results when separate tumor specimens are analyzed.

Despite the analytical feasibility of the SG-HRD test demonstrated in this study, decentralized HRD testing may not be equally feasible for all laboratories. Its cost-effectiveness depends on sample volume, sequencing capacity, batching requirements, personnel expertise and the availability of appropriate quality-control procedures. In laboratories with a low number of eligible samples, waiting to complete a sequencing batch may prolong turnaround time and reduce the expected advantages of local testing. Therefore, implementation decisions should consider not only analytical performance but also workload, infrastructure, sustainability and integration within the clinical diagnostic pathway.

Several limitations should be considered in our study. First, this pilot study included a small, selected cohort of 24 previously tested HGSOC specimens rather than a consecutive diagnostic series. Second, although the same specimens were independently analyzed at three centers, these repeated measurements do not increase the number of independent biological specimens. Third, two specimens had previous SG-HRD rather than M-HRD results. These comparisons inform consistency within the same testing platform, but do not provide independent evidence of agreement with a different assay; they were therefore excluded from the M-HRD-specific analysis. Moreover, M-HRD was used as a clinically validated comparator rather than an independent measure of biological HRD status, so agreement does not establish biological accuracy or clinical interchangeability. Finally, clinical intra-run and inter-run replication was limited to one HRD-positive specimen, supplemented by control materials, and does not establish precision across the clinically relevant range of GI values, tumor content, DNA quality, or HRD-positive and HRD-negative specimens.

In conclusion, this multicenter collaborative study supports the analytical feasibility of decentralized SG-HRD testing, with concordant *BRCA1/2* variant detection relative to previous testing and interlaboratory agreement in GI-based classification under the conditions evaluated. However, the small sample size and limited clinical replication restrict the generalizability of these findings and preclude definitive clinical validation. Implementation in routine practice requires appropriate local analytical validation, quality control, and interpretative expertise.

## 4. Materials and Methods

We included 24 HGSOC samples from three hospitals in Barcelona: (1) Hospital del Mar, (2) Hospital Universitari Vall d’Hebron, and (3) Hospital Clínic de Barcelona. Samples were previously tested as part of routine clinical care using either M-HRD or SG-HRD send-out platforms. Pathologists from each center marked the highest tumor cell regions in hematoxylin–eosin slides, ensuring at least 30% of tumor cells. Each of the three hospitals contributed eight clinical specimens. DNA samples were isolated from formalin-fixed paraffin-embedded (FFPE) tissue using either QIAamp DNA FFPE Advanced UNG Kit (QIAGEN, GmbH, Hilden, Germany), cobas DNA Sample Preparation Kit, (F. Hoffmann-La Roche AG, Basel, Switzerland) or QIAamp DNA FFPE Advanced Kit (QIAGEN). Genomic DNA quality was assessed by automated gel electrophoresis in a TapeStation 4200 system (Agilent Technologies, Santa Clara, CA, USA).

Samples included were HRD-positive due to *BRCA1*/*2* pathogenic variants, HRD-positive without detectable pathogenic *BRCA1*/2 variants, or HRR proficient according to previous results from the send-out platforms M-HRD or SG-HRD (Table 1).

**Figure 1:**
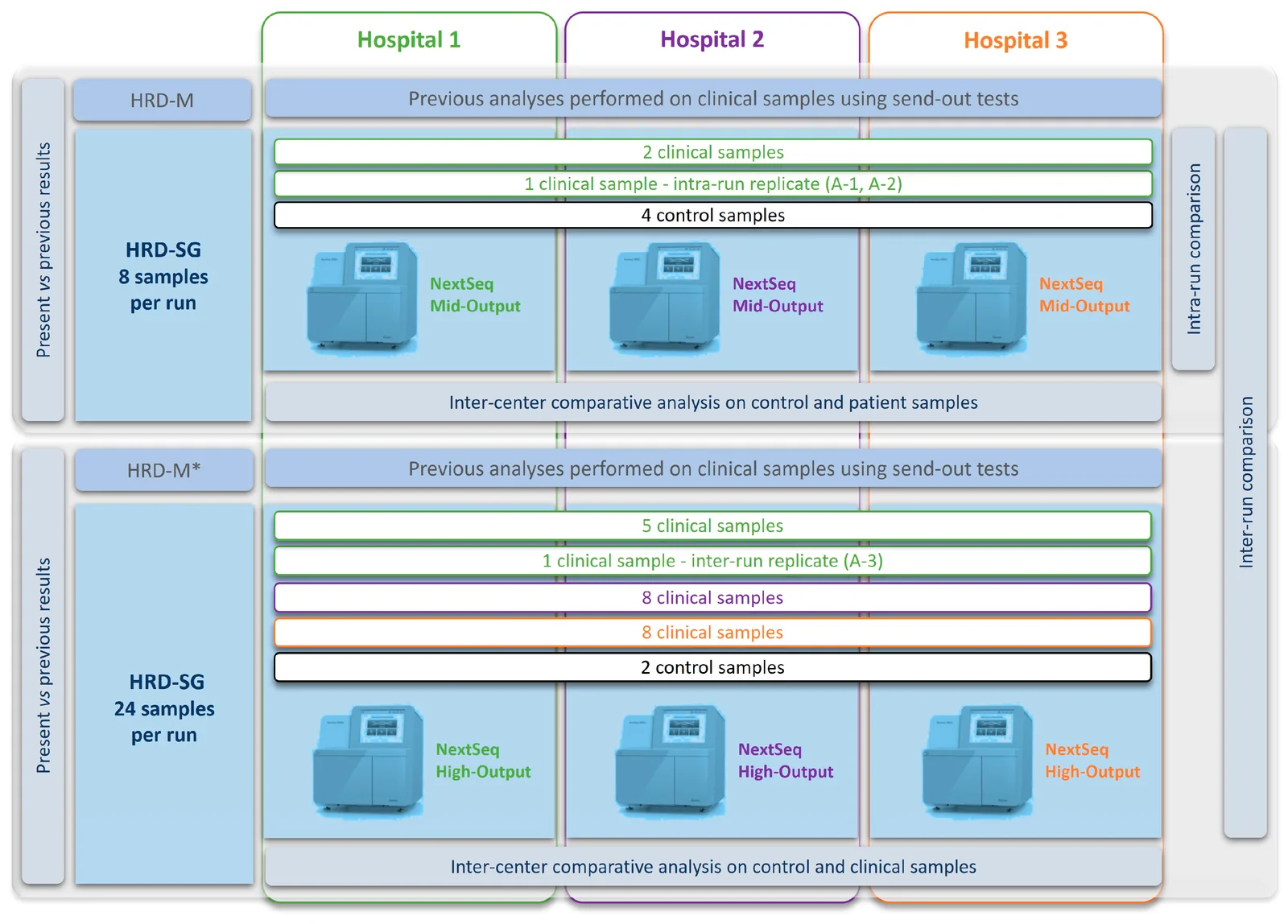
Schematic representation of the multicenter study design. Each of the three hospitals independently analyzed the 24 unique HGSOC clinical specimens using the SG-HRD workflow. Run 1 included three clinical specimens from Hospital 1, one of which was analyzed in duplicate (A-1 and A-2) for intra-run repeatability, together with four assay control materials. Run 2 included the remaining five clinical specimens from Hospital 1, the inter-run replicate (A-3) of the same clinical specimen analyzed as A-1 and A-2 in Run 1, eight specimens from Hospital 2, eight specimens from Hospital 3, together with two assay control materials. Results generated at each center were compared with previous send-out testing and across participating hospitals.

Genomic DNA libraries were prepared using the SG-HRD workflow according to the manufacturer’s instructions (SOPHiA GENETICS SA, Rolle, Switzerland). The assay combines low-pass whole-genome sequencing with targeted deep sequencing using the HRD_v1 probe panel, which covers *ATM*, *CHEK1*, *BRCA2*, *RAD54L*, *RAD51B*, *PALB2*, *FANCA*, *RAD51D*, *CDK12*, *BRCA1*, *RAD51C*, *BRIP1*, *TP53*, *BARD1*, *CHEK2*, *FANCL*, *PIK3CA*, *FGFR3*, *PPP2R2A*, *FGFR1*, *PTEN*, *FANCD2* and *NBN* genes. The sampling strategy consisted of four pools of eight samples each. Pool 1 included an HRD control library, a positive and a negative genomic stability reference sample, the SeraSeq Inherited Cancer DNA Mix control (SeraCare, Milford, MA, USA), a clinical sample in replicate (A-1 and A-2, as intra-run replicate), and two other clinical samples from hospital 1, for a total of eight samples. Pools 2, 3 and 4 each included an HRD control library (inter-run replicate), the SeraSeq Inherited Cancer DNA Mix control (SeraCare) (inter-run replicate), a clinical sample in replicate (A-3, as inter-run replicate), and 21 other clinical samples from hospitals 1, 2 and 3, for a total of 24 samples. Pool 1 was sequenced in a Mid-Output flowcell and pools 2, 3 and 4 were sequenced together using a High-Output flowcell. Each participating hospital independently performed library preparation, sequencing (NextSeq 500/550 platform (Illumina Inc., San Diego, CA, USA)), and data analysis according to the same workflow (Figure 1).

Analysis was performed on the SOPHiA DDM™ Platform. Variant detection was evaluated separately from GI-based HRD classification by comparing *BRCA1/2* calls with previous clinical results and characterized control materials. Repeatability and reproducibility experiments for variant detection used the SeraSeq Inherited Cancer DNA Mix control and one clinical specimen (sample 1). These experiments were not used to estimate sensitivity for HRD-positive/HRD-negative classification. The clinical replicate experiment was limited to one HRD-positive specimen and was interpreted only under the conditions tested.

Sequencing repeatability was determined for each pair of intra-run replicates, as the percentage of bases that were well-defined (sufficiently covered and with no low confidence variant calls) between samples among all bases well-defined in either sample. Variant repeatability was determined as the fraction of variant calls that were identical in both replicates among all evaluable variant calls identified in both replicates, using the predefined analytical criteria. Repeatability was defined as the product of sequencing and variant repeatability. Reproducibility was defined equivalently as repeatability for inter-run replicates.

For concordance analysis, the final categorical HRD classification generated by SG-HRD was first compared with the corresponding previous clinical result obtained by either SG-HRD or M-HRD. The SG-HRD GI index is reported on a continuous scale centered at 0, with values ≥ 0 classified as HRD-positive and values < 0 classified as HRD-negative, whereas M-HRD uses a genomic instability score threshold of ≥42. The GI index was analyzed as a continuous genomic-instability metric and was not itself used as the concordance endpoint. Concordance was then specifically reevaluated in samples for which a previous M-HRD result was available as the clinical comparator. Confidence intervals were calculated using Clopper–Pearson binomial CIs. Pairwise interlaboratory agreement was assessed among specimens with evaluable GI results at both laboratories. Each unique specimen was counted once per comparison. *BRCA1/2* variant detection was evaluated separately.

## Figures and Tables

**Table 1:** Sample data and results from formalin-fixed paraffin embedded clinical samples.

Sample Information				Previous Results					Present Results					
Run	Sample ID	Hospital of Origin	Tumor Content (%)	Previous Test	*BRCA* Status	*BRCA1/2* Clinically Significant Variants	HRD Status	GIS	Hospital 1		Hospital 2		**Hospital 3**	
									*BRCA1/2* Concordance	GI Index	*BRCA1/2* Concordance	GI Index	*BRCA1/2* Concordance	GI Index
1	1 ^§^	1	80	M-HRD	+	*BRCA2*: c.1308_1309del; p.(Lys437Argfs*14)	+	59	Y	5.3	Y	5.5	Y	4
1	1 ^§^	1	80	M-HRD	+	*BRCA2*: c.1308_1309del; p.(Lys437Argfs*14)	+	59	Y	4.4	Y	3.9	Y	4.7
1	7	1	80	M-HRD	−		-	27		−7.9		−7.4		−7.4
1	8	1	60	M-HRD	−		+	69		5.4		5.6		6.1
2	1 ^§^	1	80	M-HRD	+	*BRCA2*: c.1308_1309del; p.(Lys437Argfs*14)	+	59	Y	5.7	Y	4.5	Y	4.9
2	2	1	70	M-HRD	+	*BRCA1*: c.3049G>T; p.(Glu1017*)	+	82	Y	18.2	Y	19.1	Y	18.2
2	3	1	50	M-HRD	−		-	21		−13		−12.1		−12.3
2	4	1	60	M-HRD	−		NA ^#^	NA		0.6		1		0.6
2	5	1	40	M-HRD	−		-	12		−12		−12.4		−14.3
2	6	1	40	M-HRD	−		NA ^#^	35		−12.2		−11.7		NA
2	9	2	60	M-HRD	+	*BRCA2*: c.3264dup; p.(Gln1089Serfs*10)	+	53	Y	1.8	Y	1.7	Y	NA
2	10	2	70	M-HRD	−		-	21		−6.3		−7.5		NA
2	11	2	50	SG-HRD	+	*BRCA1*: c.1860del; p.(His621Metfs*5)	+	9.2 ^‡^	Y	11.5	Y	11.9	Y	10.4
2	12	2	70	M-HRD	−		+	63		5.7		5.9		6.3
2	13	2	80	SG-HRD	−		+	0 ^‡^		0		−0.2 ^¥^		−0.4 ^¥^
2	14	2	70	M-HRD	−		-	10		−11.6		−11.8		−11.7
2	15	2	40	M-HRD	+	*BRCA2*: c.2701del; p.(Ala902Leufs*2)	+	64	Y	13.1	Y	14.2	Y	12.5
2	16	2	80	M-HRD	−		+	55		4.2		5.1		4.1
2	17	3	80	M-HRD	+	*BRCA2*: c.7679_7680del; p.(Phe2560Serfs*5)	+	74	Y	6.2	Y	6.4	Y	6.6
2	18 ^†^	3	70	M-HRD	−		-	34		12		12.3		11.7
2	19	3	80	M-HRD	-		+	75		17.1		17.1		17.4
2	20	3	70	M-HRD	-		+	66		1.5		−0.7 ^¥^		−0.3 ^¥^
2	21	3	80	M-HRD	-		+	68		12.1		11.8		11.4
2	22	3	60	M-HRD	-		-	3		−16.8		−15.8		−16
2	23	3	60	M-HRD	-		-	27		−7.3		−7.9		−7.5
2	24	3	80	M-HRD	-		-	24		−13.5		−12.8		−13.1

Abbreviations: GI, genomic integrity; GIS, genomic instability score; HRD, homologous recombination deficiency; M-HRD, Myriad MyChoice^®^ CDx HRD; SG-HRD, SOPHiA DDM™ Dx HRD Solution; NA, unable to analyze; Y, yes. * Indicates a termination codon (Ter), according to HGVS nomenclature. ^§^ Clinical sample used as intra-run and inter-run control. **^†^** The sample submitted for previous M-HRD analysis was a biopsy, whereas the sample analyzed in the present study was a surgical specimen from the same patient and organ. ^‡^ Previous result obtained using SG-HRD; GI index shown. Sample 13 had a GI index of 0, corresponding to the predefined HRD positivity threshold, and was excluded from the primary concordance analysis with M-HRD. ^#^ No interpretable comparator result (Myriad HRD status rejected). ^¥^ Discordant HRD classifications associated with GI values close to the predefined positivity threshold.

## Data Availability

Derived data supporting the findings of this study are available from the PIs of each hospital, BB (Hospital del Mar Medical Research Institute), JHL (Hospital Universitari Vall d’Hebron), PJ (Hospital Clínic de Barcelona), on request.

## Supplementary Materials

The following supporting information can be downloaded at: https://www.mdpi.com/article/10.3390/ijms272110028/s1.

## Abbreviations

The following abbreviations are used in this manuscript:

AmoyDx HRD	AmoyDx^®^ HRD Focus Panel
CNAs	Copy Number Alterations
FDA	Food and Drug Administration
FFPE	Formalin-Fixed Paraffin-Embedded
GI	Genomic Integrity
HR	Hazard Ratio
HRD	Homologous Recombination Deficiency
HRR	Homologous Recombination Repair
HGSOC	High-Grade Serous Ovarian Carcinoma
LoF	Loss of Function
LOH	Loss Of Heterozygosity
LST	Large-Scale Transitions
M-HRD	Myriad MyChoice^®^ CDx HRD
MSI	Microsatellite Instability
NPA	Negative Percent Agreement
OC	Ovarian Cancer
OPA	Overall Percent Agreement
PARP	Poly(Adenosine Diphosphate–Ribose) Polymerase
PARPi	Poly(Adenosine Diphosphate–Ribose) Polymerase Inhibitors
PFS	Progression-Free Survival
PPS	Positive Percent Agreement
SG-HRD	SOPHiA DDM™ Dx HRD Solution
TAI	Telomeric Allelic Imbalance
TSO 500 HRD	TruSight™ Oncology 500 HRD
WES	Whole-Exome Sequencing
WGS	Whole-Genome Sequencing
