# Supplementary material for: Multicenter Evaluation of Decentralized Homologous Recombination Deficiency Testing for Enhanced Molecular Diagnosis of Ovarian Cancer †
Source: Int J Mol Sci. 2026 Nov 9;27(21):10028. doi: 10.3390/ijms272110028 (PMC13764358; doi:10.3390/ijms272110028)
Supplement: Supplementary file 1 [file ijms-27-10028-s001.zip › ijms-4536588-supplementary.pdf]

Table S1. Sequencing and quality metrics of all studied samples

Run	Sample ID	DNA Quality Number	Number of reads (hospital)			Percentage of mapped reads (hospital)			Percentage of BRCA coverage >100x (hospital)			Total number of fragments [M] (hospital)			WGS Fragments [M] (hospital)			Sample quality (hospital)		
			(1)	(2)	(3)	(1)	(2)	(3)	(1)	(2)	(3)	(1)	(2)	(3)	(1)	(2)	(3)	(1)	(2)	(3)
1	Control A	-	51748242	44057184	22131410	94.27%	51.53%	96.06%	100	100	100	23.5	19.9	10.3	18.9	13.8	6.9	High	High	High
1	A-1	6.22	33632036	4153258	23103128	95.01%	67.89%	94.28%	100	100	100	15.4	18.8	10.4	10.3	13.5	7.2	High	High	High
1	A-2	6.22	32429338	40681946	20000592	95.02%	67.50%	94.23%	100	100	100	14.8	18.4	9	10	13.1	6.4	High	High	High
1	Control B	8.47	46074548	40848438	18644838	94.42%	53.8%	97.01%	100	100	100	21	18.4	8.8	15.8	12.9	5.4	High	High	High
1	Control C	-	50801028	46654492	20551026	94.21%	49.28%	95.30%	100	100	100	23.1	21.4	9.5	18.2	14.2	5.4	High	High	High
1	7	5.91	32224312	40751112	16495698	93.87%	55.84%	94.65%	100	100	100	14.5	18.5	7.5	11	13.1	4.9	High	High	High
1	Control D	-	35311966	30518500	12312308	95.49%	31.79%	96.02%	100	100	100	16.5	14.2	5.8	10.1	9.7	2.9	High	High	Low
1	8	5	37702686	44364486	16510402	94.50%	63.31%	95.25%	100	100	100	17	20.1	7.5	12.3	15	5	High	High	High
2	A-3	6.22	30541178	4835584	15588418	95.51%	98.40%	98.30%	100	100	100	14.3	13.9	6.9	9.2	10.3	5.9	High	High	High
2	2	7.43	31066054	30886800	15596116	95.24%	94.09%	93.52%	100	100	100	14.4	16.9	6.5	8.9	12.8	5.5	High	High	High
2	3	6.56	31405424	37419746	14531236	94.87%	94.19%	93.84%	100	100	100	14.4	12.6	7.4	8.5	9.6	6.1	High	High	High
2	4	7.84	31441828	28011822	16437528	94.57%	93.98%	93.45%	100	100	100	22.6	15	7.3	14.6	11.5	6.2	High	High	High
2	5	7.75	49438728	33369638	16248308	94.43%	93.82%	93.48%	100	100	100	16.5	16.2	6.8	11.4	12.1	5.7	High	High	High
2	6	6.39	36287728	35986508	15195754	93.92%	93.74%	93.23%	100	100	100	15	17.7	1.4	10.4	13	0.4	High	High	Low
2	9	5.84	32654352	38682268	2936982	95.00%	94.76%	98.01%	100	100	100	14.9	13.6	3.9	10.4	10.1	2.8	High	High	Low
2	10	6.64	33572442	30872082	8816700	92.78%	92.18%	92.31%	100	100	100	16.6	14.9	1.4	11.4	10.5	0.3	High	High	Low
2	Control D	-	36887416	33024562	2965970	93.82%	93.54%	97.86%	100	100	100	14.2	2.3	7.6	9.4	0.3	0.8	High	Low	Low
2	11	6.465	37274988	42843124	39116728	93.54%	92.81%	93.79%	100	100	99.9	16.9	19.2	17.6	11.6	14.9	12.6	High	High	High
2	12	7.514	39903602	43314438	41049608	93.93%	93.28%	94.13%	100	100	100	18.1	18.7	18.7	12.3	16.4	11.5	High	High	High
2	13	8.055	40937162	45223736	43565726	94.79%	94.34%	95.03%	100	100	99.9	18.9	20.8	20.1	12	15	12.8	High	High	High
2	14	5.367	35943692	36677056	39899880	91.75%	91.68%	92.31%	100	100	100	15.8	16	17.7	11	12.4	11.7	High	High	High
2	15	5.685	36433662	34827164	40960368	92.97%	92.24%	92.51%	100	100	100	16.3	15.4	18.2	11.4	11.9	12.4	High	High	High
2	16	6.185	34424726	43267316	48104136	92.55%	92.63%	88.60%	100	100	99.9	15.3	19.2	20.2	10.6	15	14.6	High	High	High
2	17	7.67	36943568	54090040	39604616	93.82%	93.20%	93.84%	100	100	100	16.8	24.1	18.1	11.2	19.1	11.8	High	High	High
2	Control C	-	52576218	52130778	53586888	95.17%	94.37%	95.43%	100	100	100	24.3	23.9	25	15.2	17.9	13.8	High	High	High
2	18	8.3	39783474	46014450	41349086	93.39%	92.77%	93.58%	100	100	100	18	20.7	18.8	12.6	15.8	12.3	High	High	High
2	19	7.41	43834196	47685758	47279010	93.78%	92.89%	93.95%	100	100	100	20	21.6	21.7	13.6	16.6	13.8	High	High	High
2	20	7.61	35958186	50133498	43455792	94.07%	93.36%	94.25%	100	100	100	16.4	22.7	20	11.3	17.4	12.4	High	High	High
2	21	7.5	34379594	36922470	39091988	94.56%	93.87%	94.67%	100	100	100	15.7	16.8	17.9	10.7	12.9	11.7	High	High	High
2	22	7.18	37048876	51502492	35564918	94.56%	93.90%	94.76%	100	100	99.9	16.9	23.4	16.3	12.2	18.4	10.6	High	High	High
2	23	7.07	38783854	38347230	42202222	95.13%	94.50%	95.35%	100	100	100	17.9	17.6	19.6	11.9	13.5	11.9	High	High	High
2	24	6.93	34398686	41503192	39447240	94.85%	94.07%	94.75%	100	100	100	15.9	19	18.2	10.8	15	11.6	High	High	High

Total Number of DNA Fragments [M]: The total number of DNA fragments (paired-end reads) that are properly mapped. Fragments [M]: The total number of DNA fragments (paired-end reads) that contribute to the raw coverage for whole genome sequencing profile calculation. A-1, A-2 and A-3 represent replicate analyses of the same clinical specimen.
